# Socioeconomic status across the life course and smoking trajectories of older adult smokers in the U.S.

**DOI:** 10.1093/geroni/igab046.3531

**Published:** 2021-12-17

**Authors:** Jaqueline Avila, Sangah Lee, Ezinwa Osuoha, Dale Dagar Maglalang, Alexander Sokolovsky, Jasjit Ahluwalia

**Affiliations:** 1 Brown University, Providence, Rhode Island, United States; 2 Brown University, Brown University, Rhode Island, United States; 3 Cornell University, Cornell University, New York, United States

## Abstract

The objective of this study is to assess how SES over the life course impacts smoking cessation among older adult smokers in the U.S. 6,058 current smokers 50 years and older were identified from the 1998-2018 Waves of the Health and Retirement Study (HRS). The outcome of interest was smoking cessation. The main independent predictor was lifetime SES, categorized as low child and low adult SES (persistent low); low child, high adult SES; high child, low adult SES; and high child, high adult SES (persistent high). Multilevel mixed-effect logistic model was used to examine how lifetime SES predicts smoking cessation at age 65 and over time, adjusted by health and smoking covariates. The majority of older smokers had persistent high lifetime SES (60.3%), followed by high child/low adult SES (18.7%), persistent low SES (15.5%) and low child/high adult SES (5.6%). Compared to those with persistent high SES, those with persistent low SES were more likely to be Hispanic (25.9% vs. 3.0%, p<0.001) or non-Hispanic Black (22.7% vs. 8.7%, p<0.001), respectively. The adjusted results showed that at age 65, compared to those with persistent high SES, those with persistent low SES, low child/high adult SES, and low adult/high child SES were less likely to quit (OR: 0.42, 95%CI:0[.31-0.56]; OR:0.37, [0.24-0.55]; OR:0.53, [0.40-0.70], respectively). Similar results were observed over time for those with persistent low SES and low adult/high child SES. However, there was no significant difference for those with low child/high adult SES.

